# The Impact of Oral Antibiotics Prior to Cancer Diagnosis on Overall Patient Survival: Findings from an English Population-Based Cohort Study

**DOI:** 10.3390/curroncol30090614

**Published:** 2023-09-15

**Authors:** Eleni Domzaridou, Tjeerd Van Staa, Andrew G. Renehan, Natalie Cook, William Welfare, Darren M. Ashcroft, Victoria Palin

**Affiliations:** 1 National Institute for Health and Care Research Greater Manchester Patient Safety Research Collaboration, School of Health Sciences, Faculty of Biology, Medicine and Health, University of Manchester, Manchester M13 9PL, UK; darren.ashcroft@manchester.ac.uk; 2 Centre for Health Informatics, Division of Informatics, Imaging and Data Science, School of Health Sciences, Faculty of Biology, Medicine and Health, University of Manchester, Manchester M13 9PL, UK; tjeerd.vanstaa@manchester.ac.uk (T.V.S.); victoria.palin@manchester.ac.uk (V.P.); 3 Utrecht Institute for Pharmaceutical Sciences, Utrecht University, 3584 CS Utrecht, The Netherlands; 4 Centre for Health Informatics, Manchester Cancer Research Centre, University of Manchester, Manchester M13 9PL, UK; andrew.renehan@manchester.ac.uk; 5 Division of Cancer Science, School of Biology, Medicine and Health, University of Manchester, Manchester M13 9PL, UK; natalie.cook@manchester.ac.uk; 6 Christie NHS Foundation Trust, Wilmslow Road, Manchester M20 4BX, UK; 7 Public Health England Northwest, 3 Piccadilly Place, London Road, Manchester M1 3BN, UK; william.welfare@phe.gov.uk; 8 Centre for Pharmacoepidemiology and Drug Safety, Division of Pharmacy and Optometry, School of Health Sciences, Faculty of Biology, Medicine and Health, University of Manchester, Manchester M13 9PL, UK; 9 Maternal and Fetal Research Centre, Division of Developmental Biology and Medicine, University of Manchester, St Marys Hospital, Oxford Road, Manchester M13 9WL, UK

**Keywords:** antibiotics, antimicrobial resistance, cancer therapy, CPRD

## Abstract

Background: There is limited evidence in humans as to whether antibiotics impact the effectiveness of cancer treatments. Rodent studies have shown that disruption in gut microbiota due to antibiotics decreases cancer therapy effectiveness. We evaluated the associations between the antibiotic treatment of different time periods before cancer diagnoses and long-term mortality. Methods: Using the Clinical Practice Research Datalink GOLD, linked to the Cancer Registry’s and the Office for National Statistics’ mortality records, we delineated a study cohort that involved cancer patients who were prescribed antibiotics 0–3 months; 3–24 months; or more than 24 months before cancer diagnosis. Patients’ exposure to antibiotics was compared according to the recency of prescriptions and time-to-event (all-cause mortality) by applying Cox models. Results: 111,260 cancer patients from England were included in the analysis. Compared with antibiotic prescriptions that were issued in the past, patients who had been prescribed antibiotics shortly before cancer diagnosis presented an increased hazard ratio (HR) for mortality. For leukaemia, the HR in the Cancer Registry was 1.32 (95% CI 1.16–1.51), for lymphoma it was 1.22 (1.08–1.36), for melanoma it was 1.28 (1.10–1.49), and for myeloma it was 1.19 (1.04–1.36). Increased HRs were observed for cancer of the uterus, bladder, and breast and ovarian and colorectal cancer. Conclusions: Antibiotics that had been issued within the three months prior to cancer diagnosis may reduce the effectiveness of chemotherapy and immunotherapy. Judicious antibiotic prescribing is needed among cancer patients.

## 1. Introduction

The gut microbiota is a complex ecosystem composed of viruses, fungi, yeast, and bacteria, retaining a mutual balance within the host known as eubiosis [1,2,3,4,5,6]. Microbiota is dynamic and forms an ecological community in the human body that is shaped by genomic factors, diet, lifestyle, improved personal hygiene, and exposure to medication, as well as the mode of delivery at birth [7]. Microbiota composition and their related metabolites can be affected by age, concomitant medication, and environmental modifiers, and is associated with intestinal, metabolic, and neurological disorders [8,9,10]. Chemotherapy can exacerbate any pre-existing dysbiotic state (disruption to the microbiota composition) and increase gut permeability by damaging epithelial tight junctions, resulting in a loss of microbiota balance, leading to toxicity [11]. Dysbiosis is known to affect oncogenesis, cancer progression, and response to therapy [2]. It is thought that the microbiota can facilitate the mechanism of action of chemotherapeutic drugs, altering T-cell adaptive immune and inflammatory responses [12]. Earlier research has suggested several biological mechanisms through which microbiota may affect the effectiveness of chemotherapy and immunotherapy by either inhibiting chemotherapy activity [12,13], enhancing the efficacy of other chemotherapeutic drugs [13], or causing toxic side effects [14]. A recent observational study conducted on 196 patients showed that the prior use of antibiotics and immune checkpoint inhibitors may negatively affect the response to therapy and overall survival [15]. Although evidence on how microbiota could affect antitumor therapy effectiveness is largely derived from laboratory experiments, ongoing clinical trials study the effects of microbiota alterations in chemotherapy- and immunotherapy-treated patients.

Antibiotics are known to disrupt the gut microbiota [16,17] and long-term use can lead to drug resistance via the disruption of the microbiota composition [18]. Recent research involving mouse models, human tissues, and patient samples across various cancer types has shown that antibiotics can reduce the effectiveness of chemotherapy and immunotherapy by metabolising or deactivating chemotherapeutic drugs, altering immune responses, or causing mucosal damage that may lead to sepsis [1,2,3,4,5,6,7]. Furthermore, a recent observational study of 291 cancer patients revealed that antibiotic exposure within the 2 weeks before or the 6 weeks after immunotherapy may increase the risk of all-cause mortality compared to patients who do not receive antibiotics [19].

In a large population-based cohort study following patients predominantly prescribed oral antibiotics in general practice and subsequently diagnosed with cancer, we hypothesised that recently issued antibiotic prescriptions negatively affect overall mortality following cancer therapy for different cancer types. The rationale for this hypothesis was that issued antibiotics prior to cancer diagnosis could influence gut microbiota, leading to reduced effectiveness of chemotherapy and/or immunotherapy and increased mortality risk. The aim of this study was to investigate whether prior antibiotic treatment of different time periods before a cancer diagnosis is associated with all-cause mortality.

## 2. Methods

### 2.1. Data Sources and Study Design

A study cohort was generated using routinely collected, anonymised, primary care data from general practices, linked to the Cancer Registry and the Office for National Statistics (ONS) mortality records. The cohort linked primary records from English practices using the Clinical Practice Research Datalink (CPRD) GOLD to the National Cancer Registration and Analysis Service (NCRAS) of Public Health England (PHE) and the ONS. CPRD is one of the biggest databases for primary care research worldwide, including anonymised patient-related data on clinical events, referrals, laboratory test results, immunisation, and prescription details, as well as information relating to patient-level demographic information [20]. At the time of this study, approximately 600 general practices around England contributed data to CPRD GOLD, accounting for 7% of the UK population [21].

### 2.2. Study Sample

In this study, data from 2000–2017 were used from 372 practices in England with patient records that were linked to Cancer Registry and ONS data. The final cohort consisted of patients with a cancer diagnosis between 1 January 2000 and 31 December 2017. Henceforth, we refer to the date of cancer diagnosis as the index date. Children under 12 years old were excluded because microbiota diversity differs between children and adults [22] and due to limited available data. All patients were required to be registered with their general practice for at least three years before their cancer diagnosis and were then observed until death or the end of the follow-up (Figure S1).

### 2.3. Exposure and Outcome Assessment

Oral antibiotics that were prescribed to cancer patients without a previous diagnosis of cancer before the index date were analysed. Prescription records were used to delineate the exposure groups according to prior antibiotic usage, including three exposure categories: (i) recent—patients issued antibiotic prescriptions in the three months before the cancer index date; (ii) previous—patients with their last antibiotic prescription issued between 3 and 24 months prior to cancer diagnosis; and (iii) past—patients who had their last antibiotic prescription more than 24 months before their cancer diagnosis. These intervals were selected according to recent evidence from clinical trials on microbiota recovery after antibiotic exposure [15,16]. In cases of cumulative antibiotic prescriptions, the most recent prescriptions were used.

The cancer diagnoses were identified using primary care records or the Cancer Registry, with analyses conducted separately for each cohort. The cancer types of interest were selected from the primary care records based on READ codes, excluding non-melanoma skin cancer. ICD-10 codes were used to identify cancer diagnoses in the Cancer Registry (lists available at clinicalcodes.org; accessed on 1 February 2019). The cancer types of interest included the 15 most common cancer types in the UK at the time of analysis. Due to poor recording of antitumor therapy data in the Cancer Registry and an absence of cancer treatment data in the primary care records, cancers were separated into types that are predominantly treated with chemotherapy, namely leukaemia, lymphoma, and myeloma, and cancers that are predominantly treated using multiple modalities, such as surgery, radiation, chemotherapy, biological, or other types of therapies (Table S1). The outcome of interest was all-cause mortality, defined based on ONS data.

### 2.4. Covariates

Variables included in the QCancer risk tool and the Charlson Comorbidity Index (CCI) were included as potential confounders in the regression models [23]. Comorbidities that could induce microbiota alterations and loss of eubiosis (non-alcoholic fatty liver disease, inflammatory bowel disease, type 1 diabetes, rheumatoid arthritis, and ankylosing spondylitis) [24] were included. The models were adjusted for other potential confounders, including non-steroid anti-inflammatory drug (NSAID) usage three months prior to the index date [25], metformin for type 2 diabetes [26], proton pump inhibitors [27,28], and vaccination against flu and pneumococcal infections within the year before cancer diagnosis. Missingness was observed for body mass index, ethnicity, and smoking status, and so models were adjusted with binary missing indicator variables. Due to high percentages of missingness for variables such as the cancer’s stage, grade, and histology-related measurements, these variables were omitted from the adjusted regression models. Additionally, due to incomplete cancer stage data, it was not possible to stratify the analysis according to stage. In the case of patients with a late cancer diagnosis and immediate death, all patients that died within six months of the cancer index date were removed from the analysis.

### 2.5. Statistical Analysis

Cox Proportional Hazard (PH) models were developed to calculate the hazard ratio (HR) and examine whether cancer mortality is affected by the recency of antibiotic prescriptions. First, crude models were performed for each cancer type, only adjusting for age. Secondly, fully adjusted models were performed controlling for confounders, fitting one model per cancer type. The assumption for PH was checked using the Grambsch–Therneau statistical test and graphically checked by examining the scaled Schoenfeld residual plots against time, testing for zero slope. In the case of non-proportionality of the hazards over time on antibiotic prescriptions, the follow-up started 6 months after cancer diagnosis; this strategy allowed for control over bias due to early cancer mortality. In the case of a violation of the PH assumption of other predictors, standard stratification was applied, including age as a time-dependent covariate. Sensitivity analyses were conducted using general practice records to identify changes in the HR with antibiotic usage for leukaemia, lymphoma, and myeloma that were treated with chemotherapy. These analyses were stratified according to age group, sex, calendar time, and presence or absence of comorbidity based on the CCI. All of the analyses were performed using R software, version 3.5.0.

## 3. Results

A total of 111,260 cancer patients with antibiotic prescriptions were identified in CPRD GOLD, with 54,588 men with an index date between 2000 and 2017, and those aged 13–105 were assigned to three antibiotic groups as follows: 33,296 in the recent antibiotics group, 41,494 in the previous antibiotics group, and 36,470 in the past antibiotics group. Table 1 shows the baseline characteristics for patients stratified according to cancer type. For most cancers, the mean age at the date of diagnosis was over 62 years, with no differences between genders. The most common comorbidities included type 2 diabetes, chronic obstructive pulmonary disease (COPD), asthma, and renal diseases, representing more than 50% of patients in the Cancer Registry.

The findings from the primary care records and the Cancer Registry were broadly comparable. Table 2 and Table 3 and Tables S3 and S4 show that cancer patients with melanoma, breast, and prostate cancer with issued antibiotic prescriptions before cancer diagnosis had the highest one-year survival in contrast with pancreatic, lung, stomach, and oesophagus cancer patients that had the lowest one-year survival. Kaplan–Meier plots show the results from the Cancer Registry in which recent antibiotic users represent worse prognosis for leukaemia, lymphoma, myeloma, and melanoma, compared with past users. Moreover, recent antibiotic users represent worse prognosis compared with past users for all cancer types of interest, except for stomach cancer (Figures S2 and S3). The observed differences in mortality rates were statistically significant for leukaemia, lymphoma, myeloma, melanoma, lung, kidney, ovarian, pancreatic, oesophagus, uterus, colorectal, bladder, breast, and prostate cancer, but not for stomach cancer, according to the log-rank test. For recent antibiotic users who developed leukaemia or myeloma, it was observed that there was an approximately 50% decrease in overall mortality in the first two years of follow-up, while for lymphoma it was in the first four years. For cancers mainly treated with chemotherapy and/or immunotherapy, melanoma presented the highest survival percentages for the recent antibiotics group.

Table 3 summarises the results from both the crude and fully adjusted models from the primary care records and the Cancer Registry. The HR compares recent (≤3 months) and previous (3–24 months) antibiotic use to the baseline (past use; >24 months). The highest HR for chemotherapy-treated cancer patients with issued antibiotic prescriptions shortly before cancer diagnosis (recent group) was observed for leukaemia (HR 1.32, 95% CI 1.16–1.51), then for myeloma (1.19, 95% CI 1.04–1.36), and then for lymphoma (1.22, 95% CI 1.08–1.36). The HR was also high for cancer of the uterus (1.40, 95% CI 1.18–1.65), breast (1.36, 95% CI 1.23–1.49), ovarian cancer (1.34, 95% CI 1.20–1.50), and melanoma (1.28, 95% CI 1.10–1.49). The findings from the Cancer Registry suggest that the HR was lower or non-statistically significant for previous exposure than for recent antibiotic exposure; however, in the primary care records, patients who developed bladder or breast cancer and were assigned to the previous antibiotic group had a more-than-10%-higher probability of having a shorter survival in comparison to the baseline. The results for all of the cancer types examined can be found in Tables S2–S4.

Table S5 summarises the results from prescribed antibiotics in primary care, with prescription dates prior to a cancer diagnosis; thus, these antibiotics were not administered prophylactically to prevent infections. Moreover, 99.9% of these prescriptions refer to orally administered antibiotics. Amoxicillin was the most frequently prescribed antibiotic prior to cancer diagnosis. Amoxicillin had been prescribed in primary care for 31–38% of patients that developed leukaemia, lymphoma, or myeloma, followed by flucloxacillin and trimethoprim. Sensitivity analyses (Tables S6 and S7) did not reveal major changes in HRs between the different strata in both cohorts.

## 4. Discussion

This study evaluated the association between recency of oral antibiotic prescriptions that were issued from general practices and overall survival for newly diagnosed cancer patients, utilising a population-based cohort. For 11 of 15 cancers of interest, patients with issued antibiotic prescriptions up to three months before their cancer diagnosis were more likely to have a shorter survival than those who were prescribed antibiotics in the distant past. The strongest associations between recently issued antibiotic prescriptions and post-cancer diagnosis overall survival were found for leukaemia, melanoma, lymphoma, and myeloma, as well as cancer of the uterus, bladder, and breast, ovarian, and colorectal cancer. Lymphoma and myeloma are predominantly treated with chemotherapy and melanoma is predominantly treated with immunotherapy, while leukaemia treatment may include chemotherapy, transplants, and other therapies. The stratification of patients according to age, gender, calendar time, and comorbidities did not affect the direction or magnitude of these findings.

Antibiotics are known to disrupt the composition of microbiota [18]. Two literature reviews have summarised proven and potential biological pathways in which disruption to the microbiota may affect chemotherapy, immunotherapy, or radiotherapy treatment effectiveness [7,11]. Recent biological studies used mice models, human tissues, and patient stools of different cancer types to test the influence of past use of antibiotics on chemotherapy and immunotherapy by using broad-spectrum antibiotics to perturb the microbiota [12,14,29,30,31,32,33]. The key result from these studies demonstrated that antibiotics could reduce the effectiveness of chemotherapy/immunotherapy by metabolising or enhancing the enzymatic deactivation of the chemotherapeutic agent, altering the immune system responses, or by causing damage to the mucosa, leading to sepsis. The findings from a recent observational study with 291 patients with advanced cancer suggest that single or cumulative exposure to antibiotics 2 weeks prior or 6 weeks after immunotherapy may increase the risk of overall mortality for patients who receive antibiotics compared to patients who do not [19]. 

This study compared patients with histories of antibiotic prescribing prior to their cancer diagnosis. The mortality rates were compared between patients with recent, previous, and past antibiotic prescriptions. The rationale for this design was that most people are exposed to antibiotics at some point during their lifetime, restricting the possibility of a never-exposed comparator group. There is limited evidence for the duration of a wash-out period for antibiotics and the potential effects of intermittent antibiotic prescriptions on the gut microbiota. A recent investigation in healthy men who received three antibiotics for four days suggested approximate recovery to baseline microbiota composition after 1.5 months, with some bacterial species remaining undetectable 6 months after antibiotics [17]. Another study that was conducted among healthy adults who received cephalosporin showed a decrease in microbiota abundance, with the initial stage of recovery being approximately 90 days [16]. However, the effects of multidrug resistance and prolonged antibiotic usage periods, as well as the potential impact of specific antibiotic classes on gut microbiota, are subjects for further investigation to assess microbiota resilience and recovery periods. 

Our study has strengths and limitations. Firstly, we were able to replicate our results in two separate large population-based cohorts with a long follow-up. We acknowledge that the lack of randomisation is a limitation of this study. However, it is not possible to randomise newly diagnosed cancer patients to different histories of antibiotic use. On the other hand, the randomisation of patients to either an antibiotics or placebo group would require very large numbers, as only a small minority would be diagnosed with cancer shortly after the randomisation; thus, there is the possibility of residual confounding. Patients exposed to antibiotics at the date of cancer diagnosis may have a higher underlying risk of mortality, for instance due to comorbidity or the stage of cancer at presentation. Our choice of the reference group (past antibiotic use) aimed to minimise this bias, as all study patients had been exposed to antibiotics at some point. However, the lack of a negative control group of patients who were not exposed to antibiotics is a limitation. Moreover, antibiotics use before cancer diagnosis could act as a proxy for delayed diagnosis, as these patients may have been treated for what was erroneously thought to be an infectious disease, or they may have had underlying, cancer-related complications or pre-existing comorbidities requiring antibiotic therapy. The analyses were restricted to antibiotic prescriptions from general practice that were administered before cancer diagnosis; an analysis of antibiotic use after cancer diagnosis was not performed. This study also lacks data on the non-primary-care prescribing of antibiotics, including prescriptions during hospital stays, data from walk-in centres, emergency departments, and out-of-hours general practice, as these data are not available in CPRD. Moreover, there were no available data on medication adherence. Drugs such as NSAIDs and proton pump inhibitors (PPIs) can modify the gut microflora composition as well as the related metabolites and chemotherapy effectiveness [25,34,35]. However, usage of NSAIDs, PPIs and other over-the-counter drugs is not recorded in CPRD and its potential influence on mortality could not be investigated in the current study. Finally, as there are few cancers exclusively treated with surgery, it was difficult to identify and include them as negative controls in this analysis.

## 5. Conclusions

This study found that antibiotic treatment before cancer diagnosis appears to be correlated with a shorter lifespan. Patients who were prescribed antibiotic prescriptions within the three months prior to their cancer diagnosis were at a higher risk of all-cause mortality compared to those who were prescribed antibiotics in the distant past. While we cannot confirm a direct causal relationship, the findings suggest that the use of antibiotics prior to diagnosis among cancer patients should be approached with caution, ensuring that the perceived benefits outweigh the potential risks.

## Figures and Tables

**Table 1:** Baseline characteristics for cancer patients—results from interlinked Cancer Registry cohort.

	Leukaemia	Lymphoma	Myeloma	Melanoma	Ovary	Bladder	Colorectal	Uterus	Breast
Number of patients	3303	5499	2022	4701	2917	4894	15,903	3151	20,635
Age (mean) ^a^	68	66	71	62	65	74	72	67	64
Sex = female (%)	1394 (42.2)	2636 (47.9)	956 (47.3)	2607 (55.5)	2917 (100.0)	1477 (30.2)	7701 (48.4)	3151 (100.0)	20,495 (99.3)
Diabetes (%)	461 (14.0)	786 (14.3)	305 (15.1)	488 (10.4)	311 (10.7)	881 (18.0)	2765 (17.4)	593 (18.8)	2136 (10.4)
COPD (%)	546 (16.5)	942 (17.1)	338 (16.7)	682 (14.5)	454 (15.6)	869 (17.8)	2826 (17.8)	422 (13.4)	3378 (16.4)
Asthma (%)	391 (11.8)	730 (13.3)	255 (12.6)	589 (12.5)	386 (13.2)	532 (10.9)	2064 (13.0)	365 (11.6)	2844 (13.8)
CVD (%)	290 (8.8)	408 (7.4)	150 (7.4)	284 (6.0)	175 (6.0)	527 (10.8)	1462 (9.2)	158 (5.0)	1121 (5.4)
Heart failure (%)	158 (4.8)	227 (4.1)	104 (5.1)	121 (2.6)	120 (4.1)	275 (5.6)	845 (5.3)	87 (2.8)	525 (2.5)
Renal disease (%)	344 (10.4)	612 (11.1)	327 (16.2)	380 (8.1)	267 (9.2)	798 (16.3)	2027 (12.7)	353 (11.2)	1679 (8.1)

^a^ Age at the date of diagnoses, COPD: chronic obstructive pulmonary disease, CVD: cardiovascular disease.

**Table 2:** Life table based on the Cancer Registry.

Cancer Type	Time Period	Patients at Risk	Deaths	Censored	Proportion That Died	Proportion That Survived	Survival Probability	% Survival
Leukaemia	0–1 years	3303	1036	0	0.31	0.69	0.69	69%
	1–5 years	2267	835	271	0.39	0.61	0.42	42%
	5–10 years	1161	318	474	0.34	0.66	0.28	28%
Lymphoma	0–1 years	5499	1287	0	0.23	0.77	0.77	77%
	1–5 years	4212	992	628	0.25	0.75	0.58	58%
	5–10 years	2592	512	1116	0.25	0.75	0.43	43%
Myeloma	0–1 years	2022	547	0	0.27	0.73	0.73	73%
	1–5 years	1475	620	166	0.45	0.55	0.40	40%
	5–10 years	689	282	234	0.49	0.51	0.20	20%
Melanoma	0–1 years	4701	196	0	0.04	0.96	0.96	96%
	1–5 years	4505	678	768	0.16	0.84	0.81	81%
	5–10 years	3059	353	1440	0.15	0.85	0.69	69%
Ovarian	0–1 years	2917	875	0	0.30	0.70	0.70	70%
	1–5 years	2042	867	208	0.45	0.55	0.39	39%
	5–10 years	967	190	414	0.25	0.75	0.29	29%
Bladder	0–1 years	4894	1434	0	0.29	0.71	0.71	71%
	1–5 years	3460	1345	310	0.41	0.59	0.42	42%
	5–10 years	1805	534	601	0.35	0.65	0.27	27%
Colorectal	0–1 years	15,903	4068	0	0.26	0.74	0.74	74%
	1–5 years	11,835	4065	1365	0.36	0.64	0.47	47%
	5–10 years	6405	1472	2615	0.29	0.71	0.34	34%
Uterus	0–1 years	3151	299	0	0.09	0.91	0.91	91%
	1–5 years	2852	524	414	0.20	0.80	0.73	73%
	5–10 years	1914	247	825	0.16	0.84	0.61	61%
Breast	0–1 years	20,635	1197	0	0.06	0.94	0.94	94%
	1–5 years	19,438	3165	2953	0.18	0.82	0.77	77%
	5–10 years	13,320	1780	5727	0.17	0.83	0.64	64%

**Table 3:** Hazard ratios (HRs) according to cancer type from the Cancer Registry and primary care records in the CPRD, stratified according to recency of issued antibiotic prescriptions (exposure group).

		Cancer Registry		Primary Care	
Cancer Type	Exposure Group	Crude HR [95% CI]	Adjusted HR [95% CI]	Crude HR [95% CI]	Adjusted HR [95% CI]
Leukaemia	Recent	1.34 [1.17–1.52]	1.32 [1.16–1.51]	1.55 [1.37–1.75]	1.53 [1.35–1.73]
	Previous	1.12 [0.98–1.26]	1.11 [0.98–1.26]	1.10 [0.98–1.24]	1.17 [1.04–1.32]
	Past	reference	reference	reference	reference
Lymphoma	Recent	1.26 [1.12–1.41]	1.22 [1.08–1.36]	1.27 [1.16–1.40]	1.16 [1.13–1.30]
	Previous	1.13 [1.01–1.26]	1.09 [0.97–1.22]	1.07 [0.98–1.18]	1.09 [0.97–1.22]
	Past	reference	reference	reference	reference
Myeloma	Recent	1.22 [1.05–1.43]	1.19 [1.04–1.36]	1.32 [1.16–1.51]	1.14 [0.99–1.32]
	Previous	1.07 [0.93–1.24]	1.09 [0.96–1.23]	1.06 [0.94–1.19]	1.00 [0.88–1.15]
	Past	reference	reference	reference	reference
Melanoma	Recent	1.28 [1.11–1.49]	1.28 [1.10–1.49]	1.16 [1.03–1.31]	1.37 [1.21–1.56]
	Previous	1.08 [0.95–1.22]	1.08 [0.95–1.23]	1.02 [0.92–1.15]	1.07 [0.96–1.20]
	Past	reference	reference	reference	reference
Ovarian	Recent	1.40 [1.22–1.60]	1.34 [1.20–1.50]	1.26 [1.13–1.40]	1.27 [1.11–1.44]
	Previous	1.07 [0.94–1.23]	1.02 [0.92–1.14]	1.06 [0.95–1.18]	1.09 [0.96–1.24]
	Past	reference	reference	reference	reference
Bladder	Recent	1.20 [1.07–1.40]	1.39 [1.25–1.54]	1.28 [1.16–1.40]	1.34 [1.23–1.45]
	Previous	1.15 [1.00–1.30]	1.16 [1.04–1.30]	1.12 [1.01–1.23]	1.08 [0.99–1.18]
	Past	reference	reference	reference	reference
Colorectal	Recent	1.22 [1.14–1.30]	1.20 [1.13–1.28]	1.19 [1.09–1.28]	1.22 [1.14–1.30]
	Previous	1.04 [0.98–1.10]	1.03 [0.97–1.09]	1.07 [1.00–1.14]	1.05 [0.99–1.11]
	Past	reference	reference	reference	reference
Uterus	Recent	1.43 [1.22–1.69]	1.40 [1.18–1.65]	1.29 [1.06–1.57]	1.23 [1.01–1.50]
	Previous	1.14 [0.98–1.32]	1.10 [0.95–1.29]	1.07 [0.89–1.28]	1.03 [0.86–1.24]
	Past	reference	reference	reference	reference
Breast	Recent	1.49 [1.36–1.63]	1.36 [1.23–1.49]	1.39 [1.25–1.54]	1.18 [0.96–1.27]
	Previous	1.16 [1.07–1.26]	1.10 [1.01–1.20]	1.13 [1.03–1.24]	1.39 [1.27–1.51]
	Past	reference	reference	reference	reference

## Data Availability

Patient-level data from primary care were analysed from the CPRD GOLD. Public Health England provided the Cancer Registry data linked to CPRD, and the Office for National Statistics (ONS) provided the ONS data. Row data and datasets produced during the analyses are not available online and protocols to the appropriate institutions must be submitted to access the data sources.

## Supplementary Materials

The following supporting information can be downloaded at https://www.mdpi.com/article/10.3390/curroncol30090614/s1. Figure S1: Study design—definition of cohorts and antibiotic groups. Figure S2: Kaplan–Meier plots for each cancer type—English Cancer Registry (Public Health England). Figure S3: Kaplan–Meier plots—English General Practice Records (Clinical Practice Research Datalink, CPRD). Table S1: Cancer types, sequence of treatment, and possible alternative therapies [36,37]. Table S2: Baseline characteristics for cancer patients—results from interlinked Cancer Registry cohort. Table S3: Life table based on the Cancer Registry. Table S4: Hazard ratios (HRs) according to cancer type from the Cancer Registry and primary care records in the CPRD, stratified according to recency of issued antibiotic prescriptions (exposure group). Table S5: Antibiotic usage for cancer patients that received prescriptions between 0 and 3 months before cancer diagnosis. Results from the Cancer Registry cohort. Number of recent antibiotic users and percentage (%) per cancer type. Table S6: English Cancer Registry (National Cancer Registration and Analysis Service, Public Health England)—sensitivity analysis. The cohort was stratified to the following groups to test whether this classification has a significant effect on hazard ratio (HR) findings. Table S7: English General Practices (Clinical Practice Research Datalink, CPRD)—sensitivity analyses. The cohort was stratified to the following groups to test whether this classification has a significant effect on hazard ratio findings.
