# Supplementary material for: The Impact of Oral Antibiotics Prior to Cancer Diagnosis on Overall Patient Survival: Findings from an English Population-Based Cohort Study
Source: Curr Oncol. 2023 Sep 15;30(9):8434–43. doi: 10.3390/curroncol30090614 (PMC10528751; doi:10.3390/curroncol30090614)
Supplement: Supplementary file 1 [file curroncol-30-00614-s001.zip › curroncol-2557950-supplementary.pdf]

The Impact of Oral Antibiotics Prior to Cancer Diagnosis on Overall Patient Survival: Findings from an English Population-Based Cohort Study

Eleni Domzaridou [1,*], Tjeerd Van Staa [2,3], Andrew G. Renehan [4], Natalie Cook [5,6], William Welfare [7], Darren M. Ashcroft [1,8] and Victoria Palin [2,9]

[1] National Institute for Health and Care Research Greater Manchester Patient Safety Research Collaboration, School of Health Sciences, Faculty of Biology, Medicine and Health, University of Manchester, Manchester M13 9PL, UK; darren.ashcroft@manchester.ac.uk
[2] Centre for Health Informatics, Division of Informatics, Imaging and Data Science, School of Health Sciences, Faculty of Biology, Medicine and Health, University of Manchester, Manchester M13 9PL, UK; tjeerd.vanstaa@manchester.ac.uk (T.V.S.); victoria.palin@manchester.ac.uk (V.P.)
[3] Utrecht Institute for Pharmaceutical Sciences, Utrecht University, 3584 CS Utrecht, The Netherlands
[4] Centre for Health Informatics, Manchester Cancer Research Centre, University of Manchester, Manchester M13 9PL, UK; andrew.renehan@manchester.ac.uk
[5] Division of Cancer Science, School of Biology, Medicine and Health, University of Manchester, Manchester M13 9PL, UK; natalie.cook@manchester.ac.uk
[6] Christie NHS Foundation Trust, Wilmslow Road, Manchester M20 4BX, UK
[7] Public Health England Northwest, 3 Piccadilly Place, London Road, Manchester M1 3BN, UK; william.welfare@phe.gov.uk
[8] Centre for Pharmacoepidemiology and Drug Safety, Division of Pharmacy and Optometry, School of Health Sciences, Faculty of Biology, Medicine and Health, University of Manchester, Manchester M13 9PL, UK
[9] Maternal and Fetal Research Centre, Division of Developmental Biology and Medicine, University of Manchester, St Marys Hospital, Oxford Road, Manchester M13 9WL, UK
* Correspondence: eleni.domzaridou@manchester.ac.uk

Summary

For many cancer types, the survival was significantly lower for patients who received antibiotics shortly before cancer diagnosis, comparing to long past usage. Microbiome may affect cancer therapy outcomes and its potential to act as a new biomarker should be examined.

Figure S1: Study design - cohorts definition and antibiotic groups

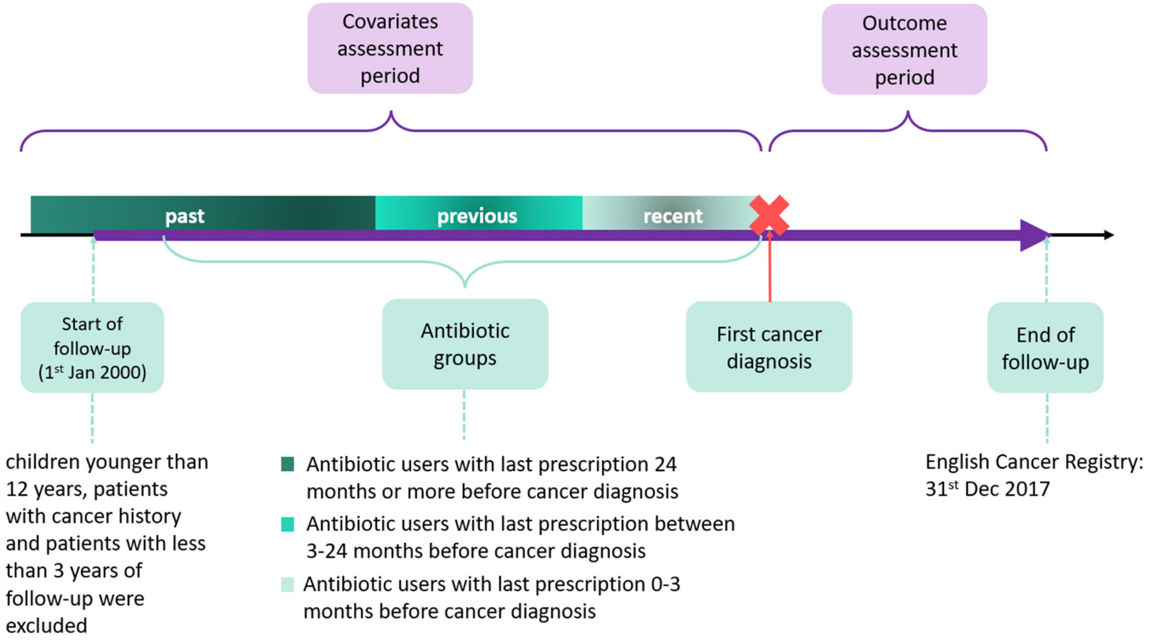

Table S1: Cancer types, sequence of treatment and possible alternative therapies.

Cancer type	Chemotherapy	Surgery	Radiotherapy	Other
Acute lymphoblastic leukaemia (ALL)	√			
Acute myeloid leukaemia (AML)	√			
Breast cancer	√ (b/a s.)	√ (1)	√ (2)	√ (3): Hormone therapy
Chronic lymphocytic leukaemia (CLL)	√	√ †		√ Targeted cancer drugs; Steroids
Chronic myeloid leukaemia (CML)	√ (2)		√	√ (1): Targeted cancer drugs; Bone marrow & stem cell transplant
Colorectal cancer	√ (b/a s.)	√ (1)	√ (2)	
Gastric cancer	√ (b/a s.)	√ (1)	√ (2)	
Hairy-cell leukaemia	√ (1)	√ (3) ‡		√ (2): Targeted cancer drugs
Head and neck cancer	√ (2)	√ (1)	√ (2)	
Hodgkin lymphoma	√		√	√ Steroids; Bone marrow transplantation
Kidney cancer	√ *	√ (1)	√ (2)	√ Cryotherapy; Arterial embolisation; Immunotherapy
Liver cancer	√	√ (1)	√	√ Transplantation
Lung cancer (small cell)	√ (1)	√ (3)	√ (2)	
Lung cancer (non-small cell)	√ (2)	√ (1)	√ (3)	
Melanoma skin cancer	√ (2)	√ (4)	√ (3)	√ (1): Biological therapy
Myeloma	√ (1)			√ (2): Steroids; Biological therapy
Non-Hodgkin lymphoma (NHL)	√	√	√	√ Targeted cancer drugs; Stem cell transplant
Oesophageal cancer	√	√ (1)	√	√ Chemoradiotherapy
Ovarian cancer	√ (b/a s.)	√ (1)		
Pancreatic cancer	√ (u)	√ (r)		
Prostate cancer	√	√	√	√ Hormone therapy; High frequency ultrasound therapy; Cryotherapy
Stomach cancer	√	√ (1)	√	√ Chemoradiotherapy
T cell childhood non-Hodgkin lymphoma (NHL)	√ (1)		√ (2)	
Testicular cancer	√ (a.s.)	√ (1)	√	√ Chemoradiotherapy
Uterus cancer	√ (2)	√ (1)	√ (2)	√ (2): Chemoradiotherapy
Urinary (bladder) cancer	√ (3)	√ (1)	√ (2)	
Vaginal cancer	√ (a. r.)	√ (1; 2)	√ (2; 1)	

† If splenectomy is needed; ‡ Splenectomy; * usually after immunotherapy/radiotherapy, b/a s.: before/after; a.r.: alongside radiotherapy; a.s.: after surgery; u: unresectable; r: respectable

Each number represents preferable treatment therapy according to clinicians' decision-making process and which depends on cancer's stage, type, spread, position, grade and patient's general health condition. Number (1) alone indicates the most usual type of therapy without excluding the possibility of other therapies. In cases where only √ exists, there are more than one different treatment options for this cancer. Neo-adjuvant and adjuvant therapies are represented by √ (b/a s.) indication. Whenever the same number occurs more than once, there are more than one alternative options. Data has been summarised from Cancer Research UK and American Cancer Society websites.[1,2]

References

1 Cancer Research UK. Your cancer type | All cancer types. 2018. http://www.cancerresearchuk.org/about-cancer/type.

2 American Cancer Society. Cancer. 2018. https://www.cancer.org/cancer/all-cancer-types.html.

Table S2: Baseline characteristics for cancer patients - results from interlinked Cancer Registry cohort.

	Leukaemia	Lymphoma	Myeloma	Melanoma	Kidney	Ovary	Bladder	Colorectal	Uterus	Breast	Prostate	Lung	Pancreas	Oesophagus	Stomach
Number of patients	3 303	5 499	2 022	4 701	3 114	2 917	4 894	15 903	3 151	20 635	17 694	17 113	3 641	3 541	3 132
Age (mean)[a]	68	66	71	62	68	65	74	72	67	64	72	72	73	72	73
Sex = Female (%)	1 394 (42.2)	2 636 (47.9)	956 (47.3)	2 607 (55.5)	1 220 (39.2)	2 917 (100.0)	1 477 (30.2)	7 701 (48.4)	3 151 (100.0)	20 495 (99.3)	0 (0.0)	7 683 (44.9)	1 977 (54.3)	1 305 (36.9)	1 151 (36.7)
Diabetes (%)	461 (14.0)	786 (14.3)	305 (15.1)	488 (10.4)	598 (19.2)	311 (10.7)	881 (18.0)	2 765 (17.4)	593 (18.8)	2 136 (10.4)	2 727 (15.4)	2 843 (16.6)	1 013 (27.8)	625 (17.7)	588 (18.8)
COPD (%)	546 (16.5)	942 (17.1)	338 (16.7)	682 (14.5)	548 (17.6)	454 (15.6)	869 (17.8)	2 826 (17.8)	422 (13.4)	3 378 (16.4)	3 032 (17.1)	5 596 (32.7)	688 (18.9)	715 (20.2)	633 (20.2)
Asthma (%)	391 (11.8)	730 (13.3)	255 (12.6)	589 (12.5)	405 (13.0)	386 (13.2)	532 (10.9)	2 064 (13.0)	365 (11.6)	2 844 (13.8)	2 149 (12.1)	2 650 (15.5)	440 (12.1)	485 (13.7)	436 (13.9)
CVD (%)	290 (8.8)	408 (7.4)	150 (7.4)	284 (6.0)	272 (8.7)	175 (6.0)	527 (10.8)	1 462 (9.2)	158 (5.0)	1 121 (5.4)	1 490 (8.4)	2 002 (11.7)	353 (9.7)	399 (11.3)	342 (10.9)
Heart failure (%)	158 (4.8)	227 (4.1)	104 (5.1)	121 (2.6)	176 (5.7)	120 (4.1)	275 (5.6)	845 (5.3)	87 (2.8)	525 (2.5)	756 (4.3)	1 114 (6.5)	179 (4.9)	235 (6.6)	216 (6.9)
Renal disease (%)	344 (10.4)	612 (11.1)	327 (16.2)	380 (8.1)	553 (17.8)	267 (9.2)	798 (16.3)	2 027 (12.7)	353 (11.2)	1 679 (8.1)	2 052 (11.6)	2 224 (13.0)	495 (13.6)	448 (12.7)	412 (13.2)

[a]age at the date of diagnoses, COPD: Chronic Obstructive Pulmonary Disease, CVD: Cardiovascular Disease

Table S3: Life table based on the Cancer Registry.

Cancer type	Time period	Patients at risk	Deaths	Censored	Proportion dying	Proportion surviving	Survival probability	% survival
Leukaemia	0-1 year	3 303	1 036	0	0.31	0.69	0.69	69%
	1-5 years	2 267	835	271	0.39	0.61	0.42	42%
	5-10 years	1 161	318	474	0.34	0.66	0.28	28%
Lymphoma	0-1 year	5 499	1 287	0	0.23	0.77	0.77	77%
	1-5 years	4 212	992	628	0.25	0.75	0.58	58%
	5-10 years	2 592	512	1 116	0.25	0.75	0.43	43%
Myeloma	0-1 year	2 022	547	0	0.27	0.73	0.73	73%
	1-5 years	1 475	620	166	0.45	0.55	0.40	40%
	5-10 years	689	282	234	0.49	0.51	0.20	20%
Melanoma	0-1 year	4 701	196	0	0.04	0.96	0.96	96%
	1-5 years	4 505	678	768	0.16	0.84	0.81	81%
	5-10 years	3 059	353	1 440	0.15	0.85	0.69	69%
Kidney	0-1 year	3 114	876	0	0.28	0.72	0.72	72%
	1-5 years	2 238	634	419	0.31	0.69	0.50	50%
	5-10 years	1 185	240	512	0.26	0.74	0.37	37%
Ovarian	0-1 year	2 917	875	0	0.30	0.70	0.70	70%
	1-5 years	2 042	867	208	0.45	0.55	0.39	39%
	5-10 years	967	190	414	0.25	0.75	0.29	29%
Bladder	0-1 year	4 894	1 434	0	0.29	0.71	0.71	71%
	1-5 years	3 460	1 345	310	0.41	0.59	0.42	42%
	5-10 years	1 805	534	601	0.35	0.65	0.27	27%
Colorectal	0-1 year	15 903	4 068	0	0.26	0.74	0.74	74%
	1-5 years	11 835	4 065	1 365	0.36	0.64	0.47	47%
	5-10 years	6 405	1 472	2 615	0.29	0.71	0.34	34%
Uterus	0-1 year	3 151	299	0	0.09	0.91	0.91	91%
	1-5 years	2 852	524	414	0.20	0.80	0.73	73%
	5-10 years	1 914	247	825	0.16	0.84	0.61	61%
Breast	0-1 year	20 635	1 197	0	0.06	0.94	0.94	94%
	1-5 years	19 438	3 165	2 953	0.18	0.82	0.77	77%
	5-10 years	13 320	1 780	5 727	0.17	0.83	0.64	64%
Prostate	0-1 year	17 694	1 306	0	0.07	0.93	0.93	93%
	1-5 years	16 388	3 424	2 629	0.23	0.77	0.72	72%
	5-10 years	10 335	2 170	4 543	0.27	0.73	0.52	52%
Lung	0-1 year	17 113	10 957	0	0.64	0.36	0.36	36%
	1-5 years	6 156	4 052	409	0.68	0.32	0.12	12%
	5-10 years	1 695	783	471	0.54	0.46	0.05	5%
Pancreas	0-1 year	3 641	2 736	0	0.75	0.25	0.25	25%
	1-5 years	905	654	51	0.74	0.26	0.07	7%
	5-10 years	200	107	37	0.59	0.41	0.03	3%
Oesophagus	0-1 year	3 541	1 927	0	0.54	0.46	0.46	46%
								continue

Cancer type	Time period	Patients at risk	Deaths	Censored	Proportion dying	Proportion surviving	Survival probability	% survival
	1-5 years	1 614	1 050	102	0.67	0.33	0.15	15%
	5-10 years	462	168	156	0.44	0.56	0.09	9%
Stomach	0-1 year	3 132	1 754	0	0.56	0.44	0.44	44%
	1-5 years	1 378	849	66	0.63	0.37	0.16	16%
	5-10 years	463	155	139	0.39	0.61	0.10	10%

Table S4: Hazards ratios (HR) according to cancer type from the Cancer Registry and Primary care records in the CPRD, stratified by recency of issued antibiotic prescriptions (exposure group).

		Cancer Registry		Primary Care	
**Cancer type**	**Exposure group**	**crude HR [95% CI]**	**adjusted HR [95% CI]**	**crude HR [95% CI]**	**adjusted HR [95% CI]**
Leukaemia	recent	1·34 [1·17-1·52]	1·32 [1·16-1·51]	1.55 [1.37-1.75]	1.53 [1.35-1.73]
	previous	1·12 [0·98-1·26]	1·11 [0·98-1·26]	1.10 [0.98-1.24]	1.17 [1.04-1.32]
	past	reference	reference	reference	reference
Lymphoma	recent	1·26 [1·12-1·41]	1·22 [1·08-1·36]	1.27 [1.16-1.40]	1.16 [1.13-1.30]
	previous	1·13 [1·01-1·26]	1·09 [0·97-1·22]	1.07 [0.98-1.18]	1.09 [0.97-1.22]
	past	reference	reference	reference	reference
Myeloma	recent	1·22 [1·05-1·43]	1·19 [1·04-1·36]	1.32 [1.16-1.51]	1.14 [0.99-1.32]
	previous	1·07 [0·93-1·24]	1·09 [0·96-1·23]	1.06 [0.94-1.19]	1.00 [0.88-1.15]
	past	reference	reference	reference	reference
Melanoma	recent	1·28 [1·11-1·49]	1·28 [1·10-1·49]	1.16 [1.03-1.31]	1.37 [1.21-1.56]
	previous	1·08 [0·95-1·22]	1·08 [0·95-1·23]	1.02 [0.92-1.15]	1.07 [0.96-1.20]
	past	reference	reference	reference	reference
Kidney	recent	1·12 [0·97-1·30]	1·11 [0·96-1·30]	1.37 [1.22-1.54]	1.32 [1.14-1.54]
	previous	0·95 [0·83-1·10]	0·94 [0·82-1·08]	1.05 [0.93-1.18]	1.07 [0.93-1.24]
	past	reference	reference	reference	reference
Ovarian	recent	1·40 [1·22-1·60]	1·34 [1·20-1·50]	1.26 [1.13-1.40]	1.27 [1.11-1.44]
	previous	1·07 [0·94-1·23]	1·02 [0·92-1·14]	1.06 [0.95-1.18]	1.09 [0.96-1.24]
	past	reference	reference	reference	reference
Bladder	recent	1·20 [1·07-1·40]	1·39 [1·25-1·54]	1.28 [1.16-1.40]	1.34 [1.23-1.45]
	previous	1·15 [1·00-1·30]	1·16 [1·04-1·30]	1.12 [1.01-1.23]	1.08 [0.99-1.18]
	past	reference	reference	reference	reference
Colorectal	recent	1·22 [1·14-1·30]	1·20 [1·13-1·28]	1.19 [1.09-1.28]	1.22 [1.14-1.30]
	previous	1·04 [0·98-1·10]	1·03 [0·97-1·09]	1.07 [1.00-1.14]	1.05 [0.99-1.11]
	past	reference	reference	reference	reference
Uterus	recent	1·43 [1·22-1·69]	1·40 [1·18-1·65]	1.29 [1.06-1.57]	1.23 [1.01-1.50]
	previous	1·14 [0·98-1·32]	1·10 [0·95-1·29]	1.07 [0.89-1.28]	1.03 [0.86-1.24]
	past	reference	reference	reference	reference
Breast	recent	1·49 [1·36-1·63]	1·36 [1·23-1·49]	1.39 [1.25-1.54]	1.18 [0.96-1.27]
	previous	1·16 [1·07-1·26]	1·10 [1·01-1·20]	1.13 [1.03-1.24]	1.39 [1.27-1.51]
	past	reference	reference	reference	reference
Prostate	recent	1·10 [1·02-1·20]	1·22 [1·15-1·30]	1.31 [1.22-1.41]	1.20 [1.13-1.28]
	previous	0·95 [0·87-1·03]	1·03 [0·97-1·10]	1.12 [1.04-1.20]	1.00 [0.95-1.06]
	past	reference	reference	reference	reference
Lung	recent	1.12 [1.05-1.21]	1.17 [1.08-1.28]	1.04 [0.97-1.12]	1.10 [1.02-1.18]
	previous	0.98 [0.92-1.06]	1.01 [0.93-1.11]	0.96 [0.89-1.04]	1.05 [0.97-1.14]
	past	reference	reference	reference	reference
Pancreas	recent	1.13 [0.98-1.30]	1.07 [0.93-1.24]	1.14 [1.05-1.23]	1.07 [0.92-1.23]

	previous	1.01 [0.88-1.14]	0.98 [0.86-1.12]	1.02 [0.95-1.11]	1.00 [0.88-1.15]
	past	reference	reference	reference	reference
Oesophagus	recent	1·09 [0·97-1·23]	1·07 [0·94-1·20]	1.06 [0.92-1.22]	1.06 [0.92-1.23]
	previous	0·96 [0·86-1·06]	0·93 [0·84-1·03]	0.98 [0.88-1.10]	0.97 [0.87-1.10]
	past	reference	reference	reference	reference
Stomach	recent	0·94 [0·82-1·08]	1·02 [0·89-1·18]	0.99 [0.86-1.14]	1.13 [1.02-1.25]
	previous	0·86 [0·77-0·96]	0·96 [0·86-1·08]	0.98 [0.88-1.10]	0.99 [0.90-1.07]
	past	reference	reference	reference	reference

Legends to figures S2-S3

Figure S2: Kaplan Meier plots for each cancer type – English Cancer Registry (Public Health England). Each curve represents the survival probability for cancer patients who used antibiotics in different time-periods before the cancer diagnosis, according to the expose definition (recent; previous; past). Recent antibiotic users had worst prognoses for all types of cancer in ten-year period except oesophagus, pancreatic and stomach cancer where it is difficult to distinguish the curves due to overlap and long-term follow-up. P-values were calculated from the Log-rank test. Confidence interval is represented in different opacity.

Figure S3: Kaplan Meier plots - English General Practice Records (Clinical Practice Research Datalink, CPRD). Each curve represents the survival probability for cancer patients who used antibiotics in different time-periods before the cancer diagnosis, according to the expose definition (recent; previous; past). Recent antibiotic users had worst prognoses for all cancers in ten-year period except lung, pancreatic and stomach cancer where it is difficult to distinguish the curves due to overlap and long-term follow-up. P-values were calculated from the Log-rank test. Confidence interval is represented in different opacity.

Figure S2

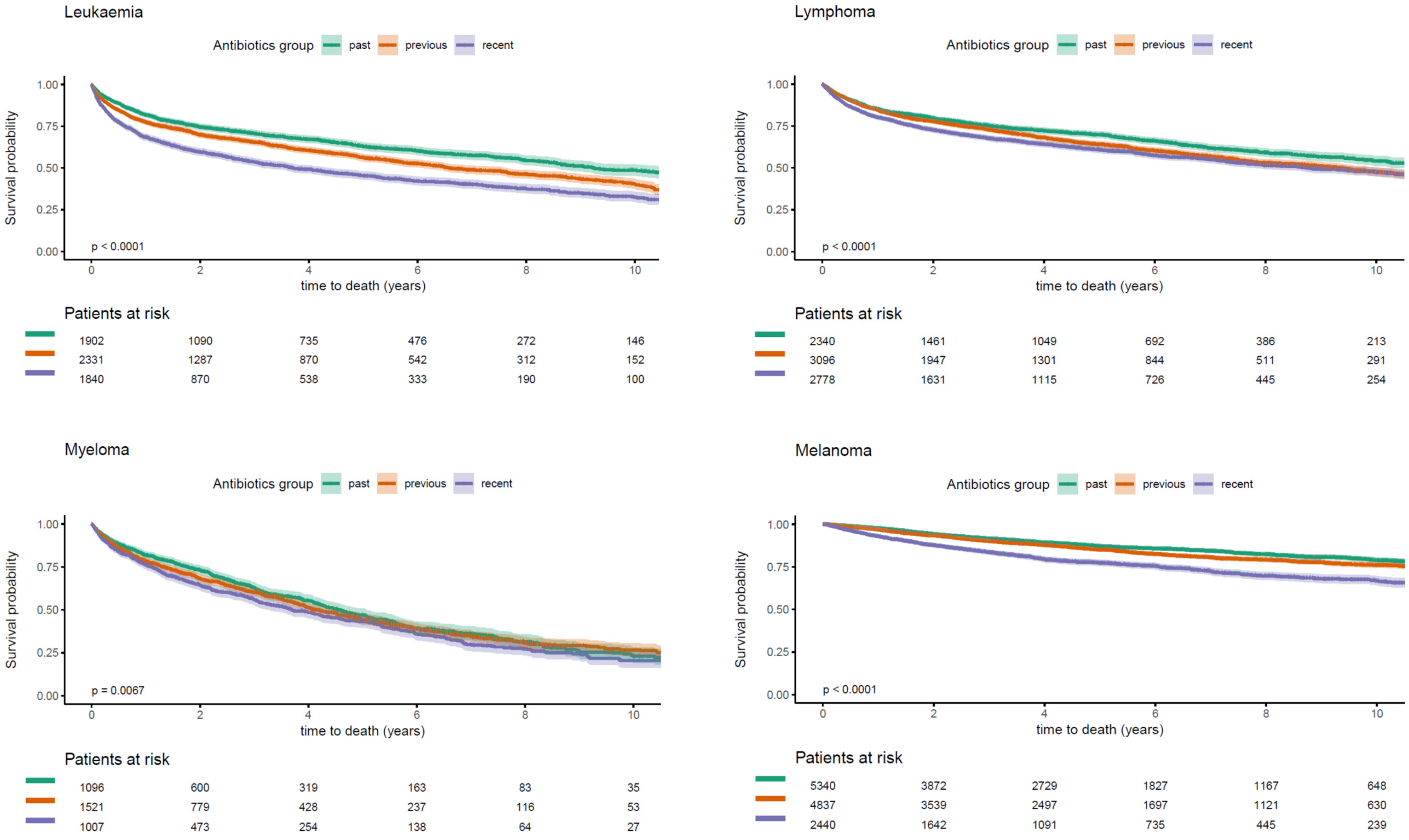
Leukaemia
Antibiotics group past previous recent
Survival probability
1.00
0.75
0.50
0.25
0.00
p < 0.0001
0 2 4 6 8 10
time to death (years)
Patients at risk
1902 1090 735 476 272 146
2331 1287 870 542 312 152
1840 870 538 333 190 100
Lymphoma
Antibiotics group past previous recent
Survival probability
1.00
0.75
0.50
0.25
0.00
p < 0.0001
0 2 4 6 8 10
time to death (years)
Patients at risk
2340 1461 1049 692 386 213
3096 1947 1301 844 511 291
2778 1631 1115 726 445 254
Myeloma
Antibiotics group past previous recent
Survival probability
1.00
0.75
0.50
0.25
0.00
p = 0.0067
0 2 4 6 8 10
time to death (years)
Patients at risk
1096 600 319 163 83 35
1521 779 428 237 116 53
1007 473 254 138 64 27
Melanoma
Antibiotics group past previous recent
Survival probability
1.00
0.75
0.50
0.25
0.00
p < 0.0001
0 2 4 6 8 10
time to death (years)
Patients at risk
5340 3872 2729 1827 1167 648
4837 3539 2497 1697 1121 630
2440 1642 1091 735 445 239

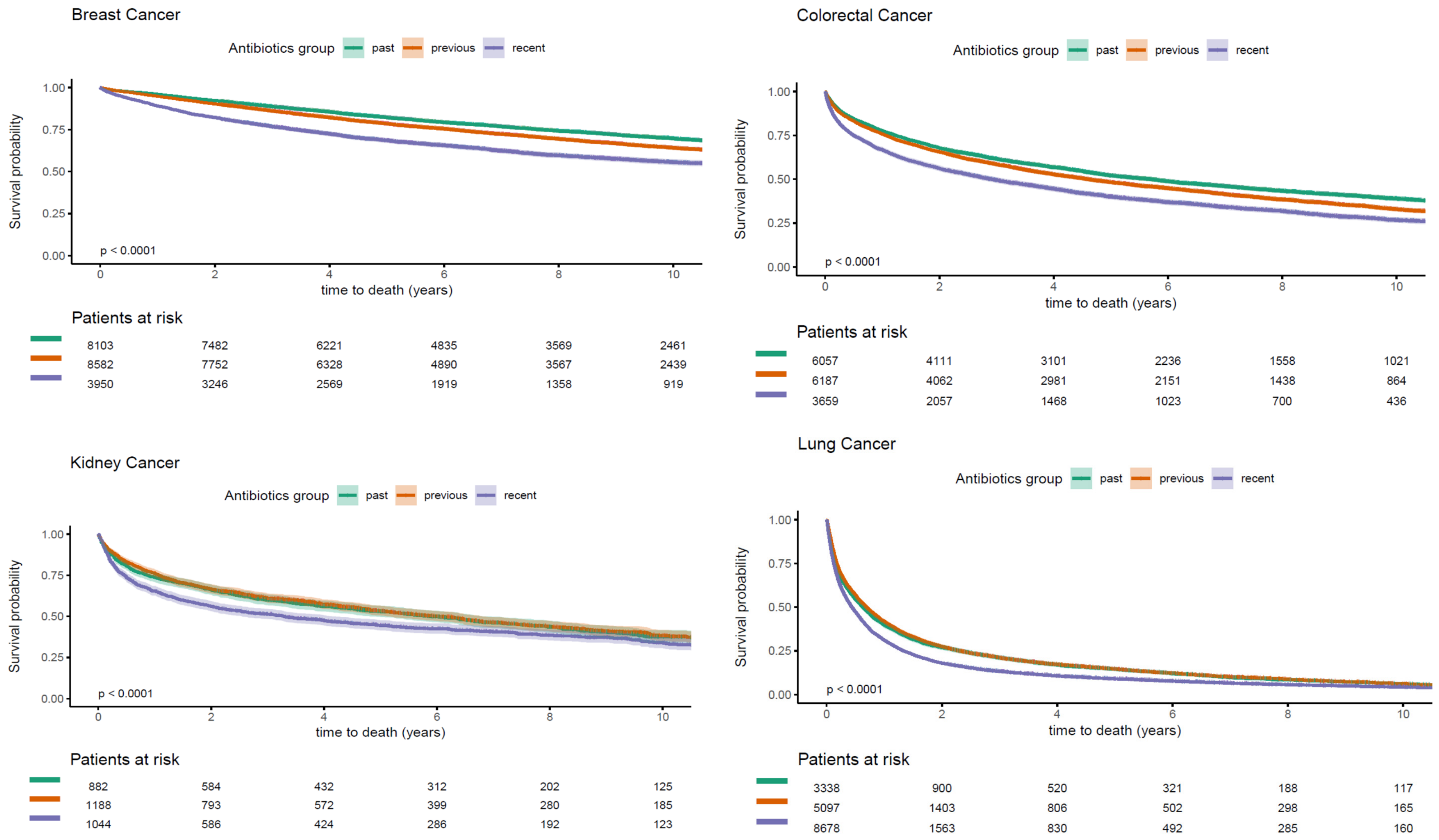
Breast Cancer
Antibiotics group past previous recent
Survival probability
1.00
0.75
0.50
0.25
0.00
p < 0.0001
0 2 4 6 8 10
time to death (years)
Patients at risk
8103 7482 6221 4835 3569 2461
8582 7752 6328 4890 3567 2439
3950 3246 2569 1919 1358 919
Colorectal Cancer
Antibiotics group past previous recent
Survival probability
1.00
0.75
0.50
0.25
0.00
p < 0.0001
0 2 4 6 8 10
time to death (years)
Patients at risk
6057 4111 3101 2236 1558 1021
6187 4062 2981 2151 1438 864
3659 2057 1468 1023 700 436
Kidney Cancer
Antibiotics group past previous recent
Survival probability
1.00
0.75
0.50
0.25
0.00
p < 0.0001
0 2 4 6 8 10
time to death (years)
Patients at risk
882 584 432 312 202 125
1188 793 572 399 280 185
1044 586 424 286 192 123
Lung Cancer
Antibiotics group past previous recent
Survival probability
1.00
0.75
0.50
0.25
0.00
p < 0.0001
0 2 4 6 8 10
time to death (years)
Patients at risk
3338 900 520 321 188 117
5097 1403 806 502 298 165
8678 1563 830 492 285 160

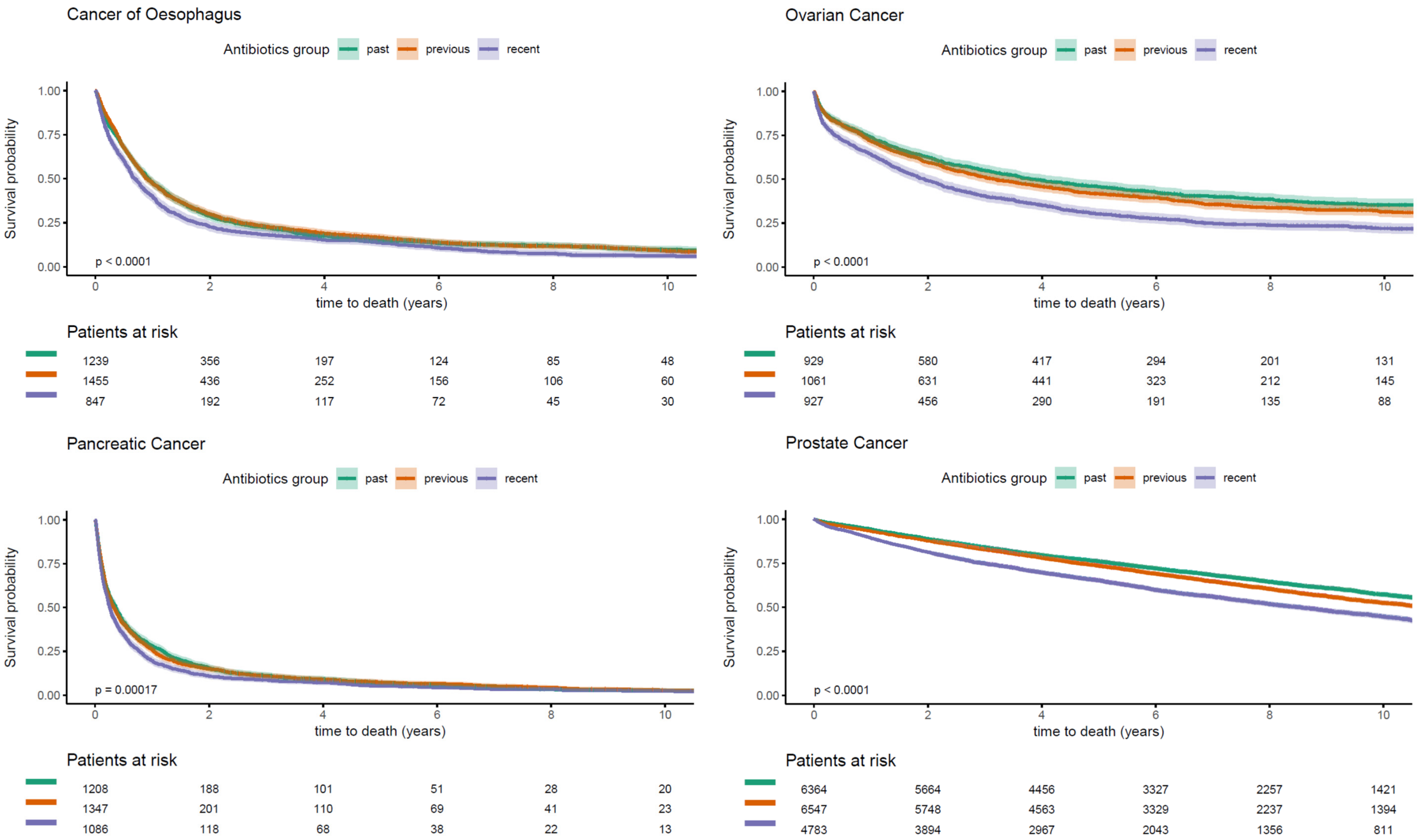
Cancer of Oesophagus
Antibiotics group past previous recent
Survival probability
1.00
0.75
0.50
0.25
0.00
p < 0.0001
0 2 4 6 8 10
time to death (years)
Patients at risk
1239 356 197 124 85 48
1455 436 252 156 106 60
847 192 117 72 45 30
Ovarian Cancer
Antibiotics group past previous recent
Survival probability
1.00
0.75
0.50
0.25
0.00
p < 0.0001
0 2 4 6 8 10
time to death (years)
Patients at risk
929 580 417 294 201 131
1061 631 441 323 212 145
927 456 290 191 135 88
Pancreatic Cancer
Antibiotics group past previous recent
Survival probability
1.00
0.75
0.50
0.25
0.00
p = 0.00017
0 2 4 6 8 10
time to death (years)
Patients at risk
1208 188 101 51 28 20
1347 201 110 69 41 23
1086 118 68 38 22 13
Prostate Cancer
Antibiotics group past previous recent
Survival probability
1.00
0.75
0.50
0.25
0.00
p < 0.0001
0 2 4 6 8 10
time to death (years)
Patients at risk
6364 5664 4456 3327 2257 1421
6547 5748 4563 3329 2237 1394
4783 3894 2967 2043 1356 811

Stomach Cancer

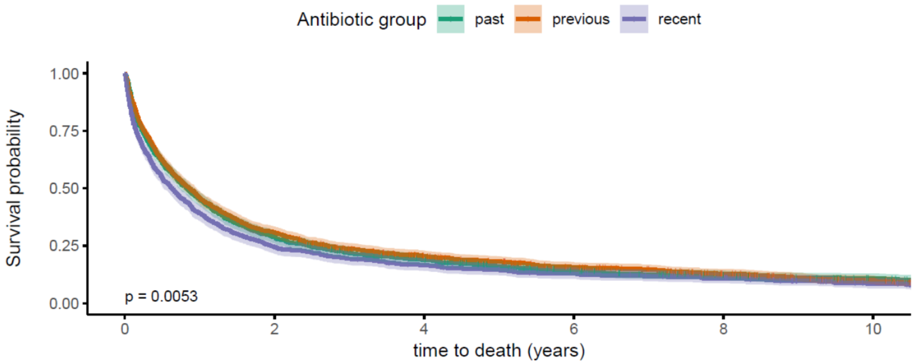

Patients at risk

1064	306	192	126	92	67
1315	405	251	173	113	73
753	184	118	82	59	29

Urinary Cancer

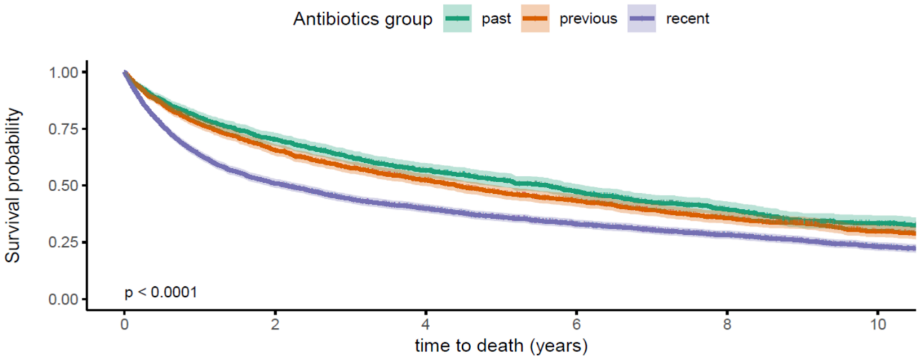

Patients at risk

987	694	520	380	249	171
1397	917	672	492	335	220
2510	1278	929	663	460	280

Cancer of Uterus

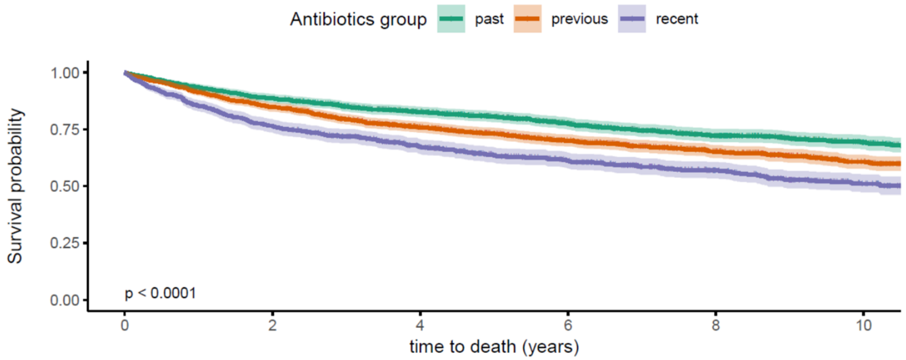

Patients at risk

1077	954	798	627	474	336
1258	1066	851	673	487	336
816	625	500	378	265	171

Figure S3

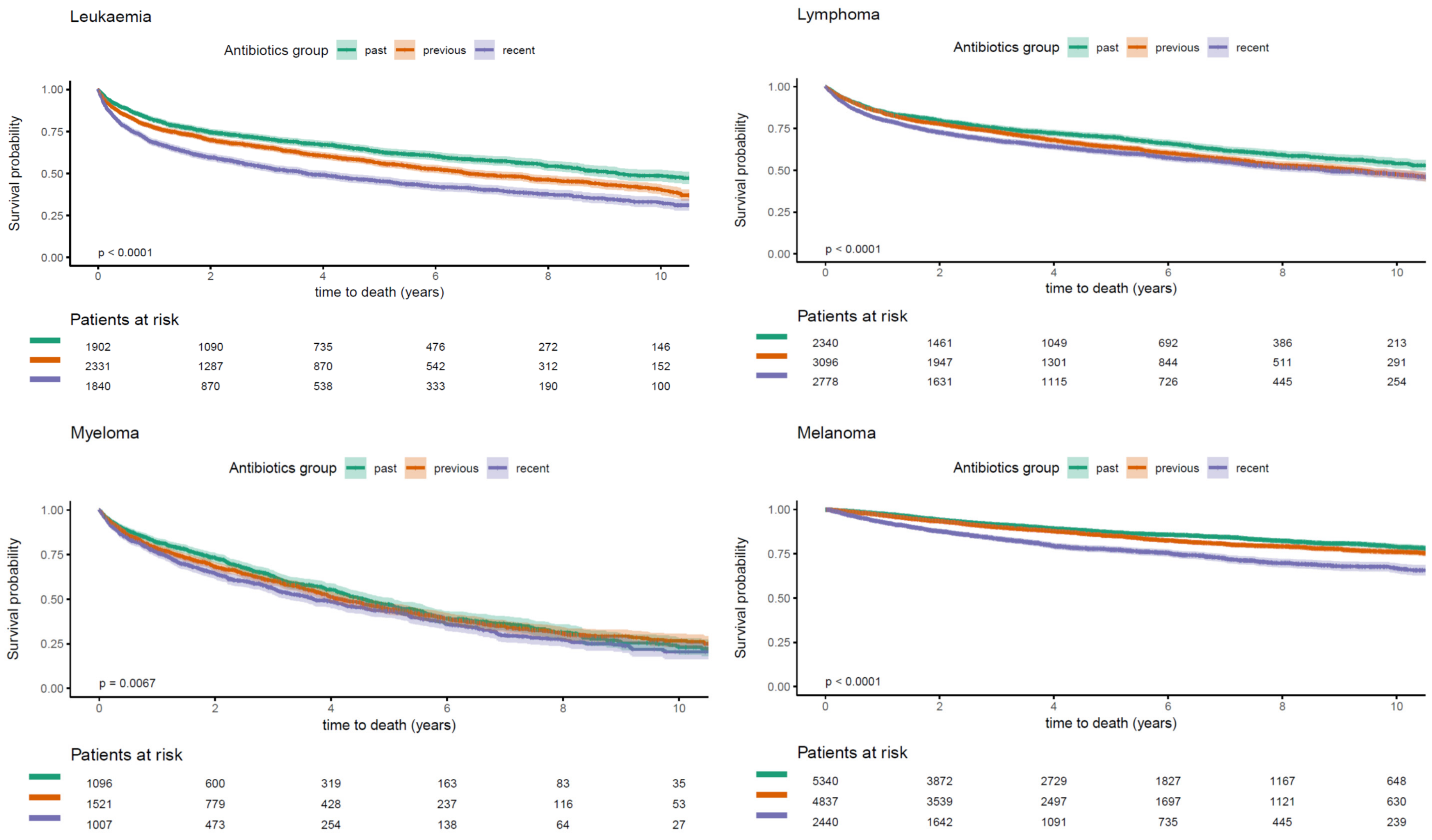
Leukaemia
Antibiotics group
past
previous
recent
Survival probability
1.00
0.75
0.50
0.25
0.00
p < 0.0001
0
2
4
6
8
10
time to death (years)
Patients at risk
1902 1090 735 476 272 146
2331 1287 870 542 312 152
1840 870 538 333 190 100
Lymphoma
Antibiotics group
past
previous
recent
Survival probability
p < 0.0001
time to death (years)
Patients at risk
2340 1461 1049 692 386 213
3096 1947 1301 844 511 291
2778 1631 1115 726 445 254
Myeloma
Antibiotics group
past
previous
recent
Survival probability
p = 0.0067
time to death (years)
Patients at risk
1096 600 319 163 83 35
1521 779 428 237 116 53
1007 473 254 138 64 27
Melanoma
Antibiotics group
past
previous
recent
Survival probability
p < 0.0001
time to death (years)
Patients at risk
5340 3872 2729 1827 1167 648
4837 3539 2497 1697 1121 630
2440 1642 1091 735 445 239

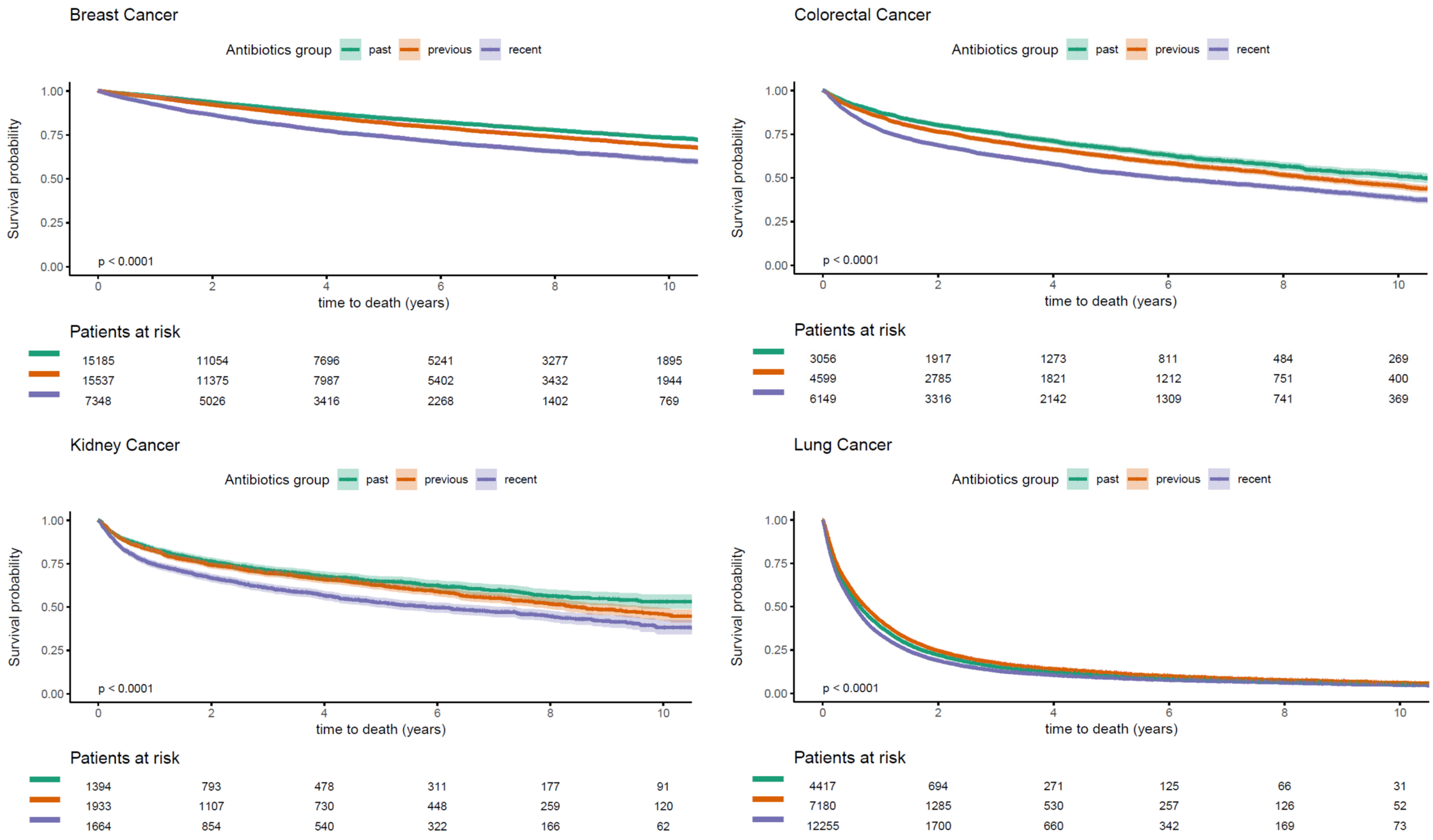
Breast Cancer
Antibiotics group past previous recent
Survival probability
1.00 0.75 0.50 0.25 0.00
p < 0.0001
0 2 4 6 8 10
time to death (years)
Patients at risk
15185 11054 7696 5241 3277 1895
15537 11375 7987 5402 3432 1944
7348 5026 3416 2268 1402 769
Colorectal Cancer
Antibiotics group past previous recent
Survival probability
1.00 0.75 0.50 0.25 0.00
p < 0.0001
0 2 4 6 8 10
time to death (years)
Patients at risk
3056 1917 1273 811 484 269
4599 2785 1821 1212 751 400
6149 3316 2142 1309 741 369
Kidney Cancer
Antibiotics group past previous recent
Survival probability
1.00 0.75 0.50 0.25 0.00
p < 0.0001
0 2 4 6 8 10
time to death (years)
Patients at risk
1394 793 478 311 177 91
1933 1107 730 448 259 120
1664 854 540 322 166 62
Lung Cancer
Antibiotics group past previous recent
Survival probability
1.00 0.75 0.50 0.25 0.00
p < 0.0001
0 2 4 6 8 10
time to death (years)
Patients at risk
4417 694 271 125 66 31
7180 1285 530 257 126 52
12255 1700 660 342 169 73

Cancer of Oesophagus

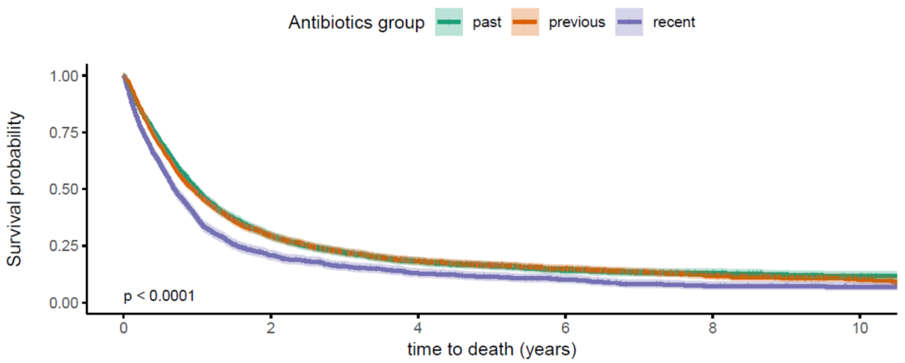

Patients at risk

past	2209	481	217	111	69	34
previous	2350	519	246	140	72	29
recent	1392	213	105	62	31	13

Ovarian Cancer

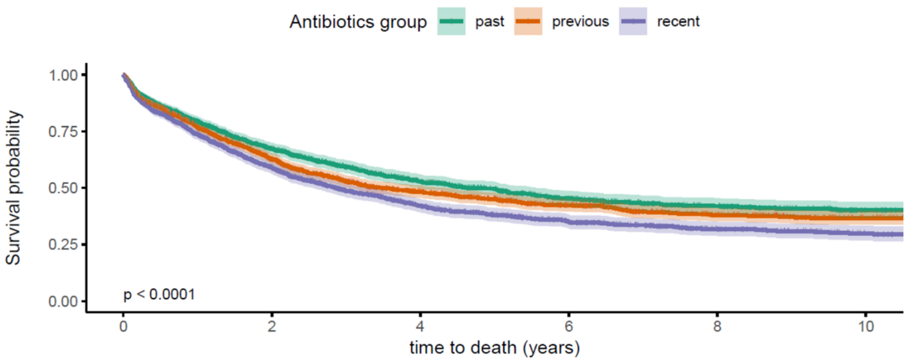

Patients at risk

past	1324	702	419	263	176	91
previous	1570	800	475	315	197	116
recent	1382	640	349	222	133	83

Pancreatic Cancer

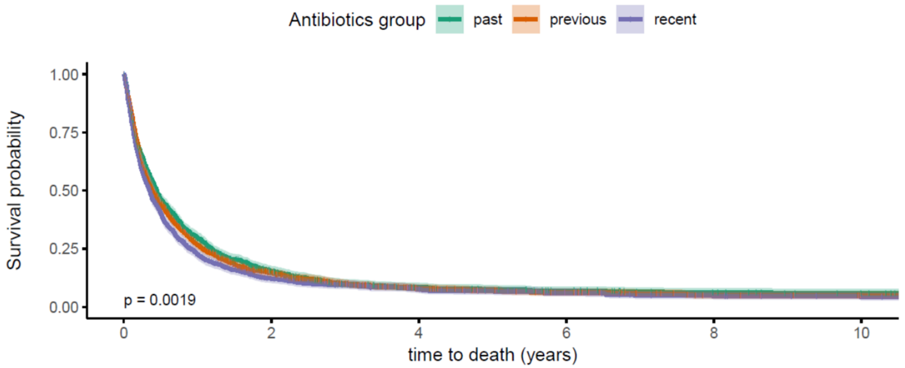

Patients at risk

past	1443	145	63	38	22	12
previous	1625	168	62	35	14	9
recent	1242	105	47	28	16	10

Prostate Cancer

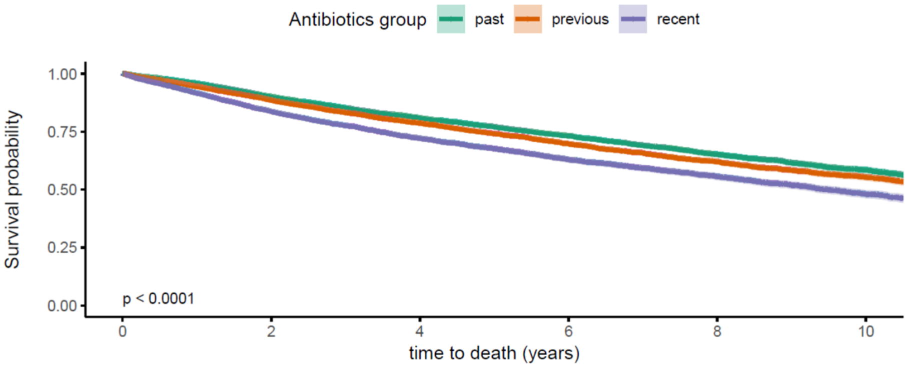

Patients at risk

past	10501	7294	4765	2990	1757	887
previous	10663	7527	5031	3183	1896	977
recent	7755	5136	3285	2034	1198	595

Stomach Cancer

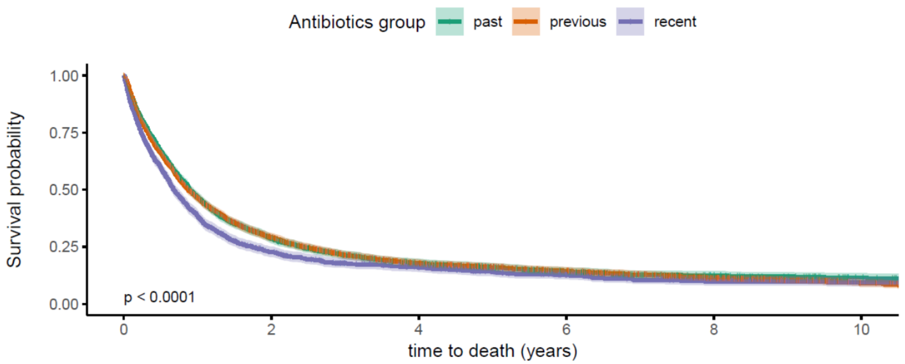

Patients at risk

2296	477	230	128	78	36
2564	583	281	171	92	40
1477	260	142	81	41	23

Urinary Cancer

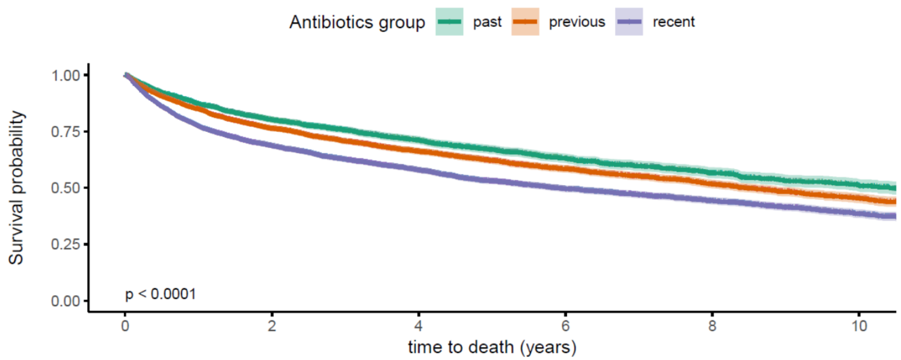

Patients at risk

3056	1917	1273	811	484	269
4599	2785	1821	1212	751	400
6149	3316	2142	1309	741	369

Cancer of Uterus

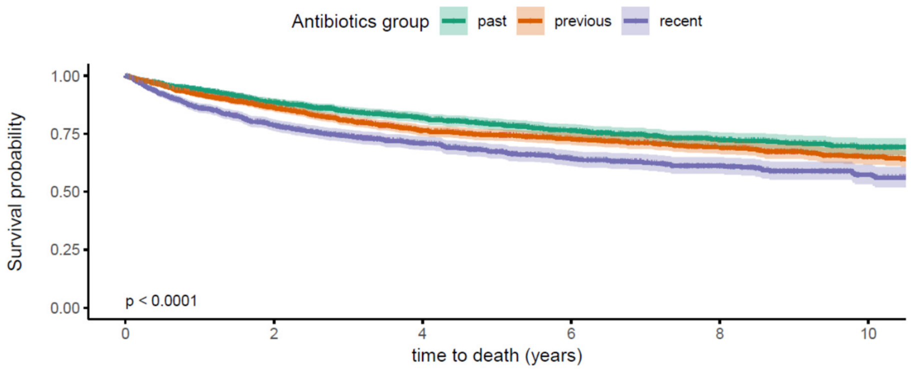

Patients at risk

1470	998	676	444	254	138
1673	1133	733	504	323	172
1179	732	473	308	180	98

Table S5: Antibiotics usage for cancer patients that received prescriptions between 0-3 months before cancer diagnosis. Results from the Cancer Registry cohort. Number of recent antibiotic users and percentage (%) per cancer type.

**Antibiotics***	**Leukemia**	**Lymphoma**	**Myeloma**
Amoxicillin (b)-amoxicillin/Ca†	397 (38)	703 (38)	231 (41)
Flucloxacillin (b)	110 (11)	220 (12)	44 (8)
Trimethoprim (S)	85 (8)	149 (8)	74 (13)
Erythromycin (M)	83 (8)	127 (7)	27 (5)
Clarithromycin (M)	78 (7)	139 (7)	41 (7)
Phenoxymethylpenicillin (b)	62 (6)	102 (5)	11 (2)
Cephalexin (C)	53 (5)	86 (5)	35 (6)
Doxycycline (T)	52 (5)	87 (5)	24 (4)
Ciprofloxacin (Q)	48 (5)	93 (5)	29 (5)
Cefaclor (C)	18 (2)	17 (1)	9 (2)
Metronidazole (Ni)	15 (1)	35 (2)	4 (1)
All antibiotic users	1043 (100)	1864 (100)	570 (100)

*Antibiotic route may include oral, intramuscular, intravenous and rectal administration however, 99.9% refer to oral antibiotics; †Ca: Clavulanic acid; Antibiotic categories in parentheses: (b) = b-lactam; (S) = Sulphonamides; (M) = Macrolides; (C) = Cephalosporins; (Q) = Quinolones; (T) = Tetracyclines; (Ni) = Nitroimidazole

Table S6: English Cancer Registry (National Cancer Registration and Analysis Service, Public Health England) - sensitivity analysis. The cohort was stratified to the following groups to test whether this classification has a significant effect on hazards ratio (HR) findings.

		Leukaemia	Lymphoma	Myeloma
**Stratification by**	**antibiotics group**	**HR [95% CI]**	**HR [95% CI]**	**HR [95% CI]**
people younger than 65 years	recent	1.70 [1.30-2.22]	1.29 [1.05-1.60]	1.27 [0.94-1.70]
	previous	1.38 [1.04-1.81]	1.12 [0.90-1.39]	1.24 [0.94-1.62]
	past	reference	reference	reference
people 65 years and older	recent	1.15 [0.96-1.37]	1.06 [0.94-1.21]	1.18 [0.99-1.41]
	previous	1.13 [0.96-1.36]	1.06 [0.93-1.22]	1.01 [0.86-1.19]
	past	reference	reference	reference
gender = male	recent	1.28 [1.08-1.52]	1.18 [1.02-1.38]	1.10 [0.89-1.66]
	previous	1.16 [1.00-1.36]	1.10 [0.95-1.28]	1.08 [0.89-1.31]
	past	reference	reference	reference
gender = female	recent	1.35 [1.10-1.67]	1.13 [0.96-1.34]	1.34 [1.07-1.67]
	previous	1.14 [0.94-1.39]	1.16 [0.99-1.36]	1.09 [0.88-1.35]
	past	reference	reference	reference
follow-up before 2008	recent	1.15 [0.97-1.38]	1.03 [0.87-1.21]	0.88 [0.66-1.18]
	previous	1.08 [0.91-1.27]	1.20 [1.03-1.40]	0.76 [0.57-1.00]
	past	reference	reference	reference
follow-up after 2008	recent	1.21 [1.00-1.47]	1.19 [0.98-1.45]	1.11 [0.87-1.41]
	previous	1.49 [1.22-1.81]	1.06 [0.88-1.29]	1.06 [0.84-1.32]
	past	reference	reference	reference
people without comorbidities*	recent	1.23 [1.01-1.50]	1.06 [0.87-1.29]	1.32 [1.04-1.68]
	previous	1.34 [1.10-1.66]	1.11 [0.92-1.33]	1.16 [0.93-1.44]
	past	reference	reference	reference
people with comorbidities*	recent	1.15 [1.00-1.33]	1.11 [0.97-1.29]	1.09 [0.89-1.33]
	previous	1.58 [1.36-1.83]	1.02 [0.89-1.18]	1.00 [0.82-1.21]
	past	reference	reference	reference

*People who did not have any recorded comorbidity according to either Charlson Comorbidity Index or QCancer risk tool related variables on which the models were adjusted.

Table S7: English General Practices (Clinical Practice Research Datalink, CPRD) - sensitivity analyses. The cohort was stratified to the following groups to test whether this classification has a significant effect on hazards ratio findings.

		**Leukaemia**	**Lymphoma**	**Myeloma**
**Stratification by**	**antibiotics group**	**HR [95% CI]**	**HR [95% CI]**	**HR [95% CI]**
people younger than 65 years	recent	2.04 [1.32-3.35]	1.94 [1.08-3.83]	1.14 [0.87-1.49]
	previous	1.52 [0.91-2.71]	0.98 [0.61-1.88]	1.10 [0.86-1.39]
	past	reference	reference	reference
people 65 years and older	recent	1.35 [1.17-1.56]	1.12 [0.98-1.29]	1.06 [0.92-1.22]
	previous	1.05 [0.92-1.20]	1.10 [0.96-1.25]	0.93 [0.81-1.05]
	past	reference	reference	reference
gender = male	recent	1.68 [1.44-1.96]	1.2 [1.06-1.36]	1.13 [0.95-1.34]
	previous	1.16 [1.00-1.35]	1.04 [0.92-1.18]	0.99 [0.85-1.16]
	past	reference	reference	reference
gender = female	recent	1.36 [1.12-1.66]	1.26 [1.09-1.45]	0.99 [0.82-1.18]
	previous	1.07 [0.88-1.29]	1.11 [0.96-1.28]	0.92 [0.78-1.09]
	past	reference	reference	reference
follow-up before 2008	recent	1.43 [1.22-1.69]	1.21 [1.06-1.38]	1.08 [0.91-1.29]
	previous	1.12 [0.96-1.31]	1.09 [0.96-1.24]	0.97 [0.83-1.14]
	past	reference	reference	reference
follow-up after 2008	recent	1.65 [1.38-1.99]	1.30 [1.13-1.49]	0.99 [0.79-1.23]
	previous	1.12 [0.93-1.33]	1.01 [0.88-1.16]	0.93 [0.76-1.14]
	past	reference	reference	reference
people without comorbidities*	recent	1.72 [1.47-2.02]	1.18 [1.01-1.37]	1.11 [0.91-1.36]
	previous	1.06 [0.91-1.25]	1.12 [0.97-1.30]	0.95 [0.80-1.14]
	past	reference	reference	reference
people with comorbidities	recent	1.46 [1.24-1.71]	1.34 [1.19-1.51]	1.12 [0.96-1.30]
	previous	1.12 [0.96-1.30]	1.05 [0.93-1.18]	1.03 [0.89-1.19]
	past	reference	reference	reference

*People who did not have any recorded comorbidity according to either Charslon Comorbidity Index or QCancer risk tool related variables on which the models were adjusted.
